# The Impact of the Covid-19 Pandemic on Food Consumers' Awareness of Antimicrobial Resistance, OneHealth, and Animal Welfare Information on Food Labels

**DOI:** 10.3389/fvets.2021.678509

**Published:** 2021-06-29

**Authors:** Áine Regan, Sharon Sweeney, Claire McKernan, Tony Benson, John Hyland, Moira Dean

**Affiliations:** ^1^Department of Agri-food Business and Spatial Analysis, Teagasc Mellows Campus, Athenry, Ireland; ^2^Faculty of Medicine, Health and Life Sciences, The Institute for Global Food Security, Queen's University Belfast, Belfast, United Kingdom

**Keywords:** antimicrobial resistance, antibiotics, consumer behaviour, COVID-19, food choice, labelling

## Abstract

Covid-19 is a OneHealth crisis with far-reaching and unexpected impacts on many aspects of society. Previous OneHealth issues, such as antimicrobial resistance (AMR), have not received a similar level of attention or action from the public despite representing significant public health and economic threats to society. The current study aimed to explore whether the Covid-19 pandemic may act as a catalyst to increase public awareness related to OneHealth issues, in particular, AMR. This short paper presents overview findings from a survey carried out in September 2020 with a representative sample of food consumers on the island of Ireland (*n* = 972). The survey revealed Covid-19 had increased awareness of AMR amongst 47% of respondents; increased awareness of connected animal and human health amongst 43% of respondents; and increased awareness of animal welfare information on food labels amongst 34% of respondents. A cluster analysis revealed five distinct consumer segments impacted differently by Covid-19. These segments differed in their levels of objective and subjective knowledge of antibiotic use practises in farming, AMR risk perception, and attributions of responsibility for action on AMR. Findings are discussed with respect to future efforts by the agri-food sector to communicate with the public about AMR and responsible antibiotic use in farming, with particular emphasis on the implications for strategies that incorporate front-of-pack labelling.

## Introduction

With evidence strongly supporting Covid-19 as a zoonotic, “animal to human disease,” this pandemic reinforces the “OneHealth” concept and the interconnectedness of human and animal health (1). Covid-19 is the most globally and societally impactful infectious disease to emerge at the human-animal interface. However, it is only one of many examples emphasising the links between human and animal health. For example, antimicrobial resistance (AMR) is a OneHealth emergency with equal, if not more, health and economic impacts (2). Antimicrobial resistance could be aggravated further as a result of Covid-19, with healthcare workers turning to antimicrobials to treat Covid-19 patients (2, 3).

Antimicrobial resistance is exacerbated by human behaviours that lead to overuse and misuse of antibiotics in human and animal health and the contamination of the environment with antibiotics. On the island of Ireland, a OneHealth approach frames the action plans of both governments to tackle AMR (4, 5). In the agricultural sector, incoming EU regulations in 2022 will target the habitually and culturally ingrained practises of using antibiotics for disease prevention in livestock and treating animals with antibiotics that are deemed critically important for human medicine (6, 7). Efforts have been intensifying to change farm-level practises in line with these regulations. Attention has also focused on how best to communicate with the public on this topic and to consider the role of market-level strategies to facilitate the systemic changes that are required (e.g., food labels and quality assurance schemes) (7, 8). On the island of Ireland, there are no quality assurance schemes which communicate *specifically* about antibiotic use and farm animal welfare on food labels to consumers. Front-of-pack labels require a baseline level of awareness and understanding by the consumer, and research suggests public awareness about antibiotic use on farms is low (9). Consumers are also not a homogenous group, and motivation to engage with such labels will vary. Furthermore, there are specific concerns around absence labelling schemes (e.g., “*antibiotic-free”*; “*no antibiotics ever”*) which fail to communicate the complexities of responsible antibiotic use in agriculture—on the one hand, agriculture needs to reduce its' use of antimicrobials, but to eliminate them completely could lead to unintended impacts on farm animal welfare (10, 11). Such authors have instead argued the need for a more nuanced labelling approach that communicates “responsible antibiotic use.” Moving beyond labelling, it has been argued that transformational changes are needed in the food supply chain to forge greater links between consumers and food producers (12) To explore how best to communicate with the public on this topic, research is first required to understand consumers' perceptions of AMR, OneHealth, and antibiotic use in agriculture.

As we have seen previously with concerns around agricultural practises and environmental sustainability (13), if AMR becomes a social issue of increased importance to the public, it is likely consumers will form strong opinions on agricultural antibiotic practises which could shape their purchasing and consumption decisions. Given its inherent OneHealth nature, it is worth considering what role the Covid-19 pandemic has played in catalysing consumers in this respect. Covid-19 has brought about unprecedented change in the attitudes and behaviours of humans in many areas of daily life. With respect to food, studies have already demonstrated a Covid-19 impact on food choice and purchasing habits (14, 15); cooking skills (16); and food waste management (17). The current study was interested to understand whether this effect has extended specifically to increasing consumers' awareness of OneHealth issues such as AMR; their awareness of connected animal and human health; and their awareness of animal welfare information on food packaging labels.

## Materials and Methods

### Survey Participants and Procedure

A cross-sectional online survey was developed to explore consumer perceptions of farm animal welfare, AMR, and agricultural antibiotic use. To pilot the survey, cognitive one-to-one interviews were carried out with a convenience sample of the target population (*n* = 9). Participants were encouraged to “think aloud” as they read and responded to survey items. This is a proven method for identifying areas of confusion or misinterpretation, and increasing face validity and relevance (18).

The survey was refined and administered to food consumers in the Republic of Ireland and Northern Ireland (*n* = 972) during September 2020. A market research agency recruited participants and administered the survey. A quota sampling procedure achieved a sample representative of gender, age, region (urban/rural) and social class for both regions. Inclusion criteria included: being aged 18+; being a consumer of either meat or dairy; and being at least partially responsible for household grocery shopping. Individuals holding the occupation of “farmer” were excluded from recruitment. Details of the socio-demographic breakdown of the sample are provided in Supplementary File 1. Participants completed the survey online, which took approximately 10–15 min. Informed consent was obtained at the outset of the survey and a full debrief on AMR and antibiotic use in agriculture was provided upon completion.

Survey variables used in the current study included: (1) Perceived impact of Covid-19 on awareness of AMR, OneHealth, and animal welfare food labels; (2) AMR risk perception; (3) Objective (actual) knowledge of antibiotic use in farming; (4) Subjective (perceived) knowledge of antibiotic use in farming; (5) Attributions of responsibility for AMR and; (6) Socio-demographics. Detailed information on wording, scoring, and source of all variables can be found in Supplementary File 2.

### Data Analyses

A cluster analysis was carried out using the mean perception scores from three items measuring the perceived impact of Covid-19 on awareness of (1) AMR; (2) OneHealth, and; (3) information about animal welfare on food labels. Before beginning the cluster analysis, variables were examined for collinearity. Examination of the correlation matrix found no substantial multicollinearity between the cluster variates (*r* <0.6 in all cases) and the variance inflation factor (VIF) scores were all less than the recommended cut-off of 10 (range: 0.55–1.83). A hierarchical cluster analysis using Ward's minimum variance method was carried out to determine the optimal number of segments. Squared Euclidean distance was selected as the distance measure. The optimal cluster solution was selected following an investigation of the agglomeration schedule and a visual inspection of the dendrogram. The 3-, 4-, and 5-cluster solutions were profiled against the cluster variates. The profiling demonstrated that the five-cluster solution provided the most distinct and conceptually meaningful clustering of participants (Table 1). Based on recommended guidelines (19), a z-score of more than ±0.5 was used to identify the distinctive characteristics of each cluster and to assist in the subjective task of labelling the clusters. One-way ANOVA's and chi-squared tests were used to compare the clusters across key profiling variables.

**Table 1:** Means and standard deviations for the Covid-19 impact variables across the five clusters.

**Impact of Covid-19**	**Cluster 1**	**Cluster 2**	**Cluster 3**	**Cluster 4**	**Cluster 5**	**Total**
	**Unaware**	**Semi-aware**	**AMR/OneHealth aware**	**OneHealth unaware**	**Highly aware**	
	*n* = 161 (16%)	*n* = 288 (30%)	*n* = 201 (21%)	*n* = 119 (12%)	*n* = 203 (21%)	***n*** **=** **972**
Increased awareness of AMR^a^	2.06 (0.74)	2.76 (0.49)	3.74 (0.67)	4.05 (0.22)	4.27 (0.45)	**3.32 (0.97)**
	*z =* –*1.30*	*z =* –*0.58*	*z = 0.43*	*z = 0.75*	*z = 0.98*	
Increased awareness of connected animal-human health^a^	1.76 (0.46)	3.20 (0.45)	3.94 (0.56)	2.82 (0.40)	4.30 (0.46)	**3.30 (0.97)**
	*z =* –*3.35*	*z =* –*0.10*	*z = 0.66*	*z =* –*0.50*	*z = 1.03*	
Increased awareness of animal welfare information on food labels^a^	2.07 (0.85)	3.18 (0.60)	2.51 (0.63)	3.45 (0.63)	4.28 (0.45)	**3.12 (0.98)**
	*z =* –*1.07*	*z = 0.10*	*z =* –*0.62*	*z = 0.34*	*z = 2.58*	

a *Range: strongly disagree (1) to strongly agree (5)*.

## Results and Discussion

Almost half (46.7%) of participants indicated that they agreed (38.1%) or strongly agreed (8.6%) with the statement that as a result of the Covid-19 pandemic, “*I am now more aware of antibiotic resistance*.” 42.9% of the participants indicated that they agreed (33.4%) or strongly agreed (9.5%) with the statement “*I am now more aware of the connection between the management of animal health and impact on human health*.” Finally, one in three (33.8%) indicated they agreed (26.3%) or strongly agreed (7.5%) with the statement “*I look more at the labelling on food products for animal welfare information*.”

**Figure 1:**
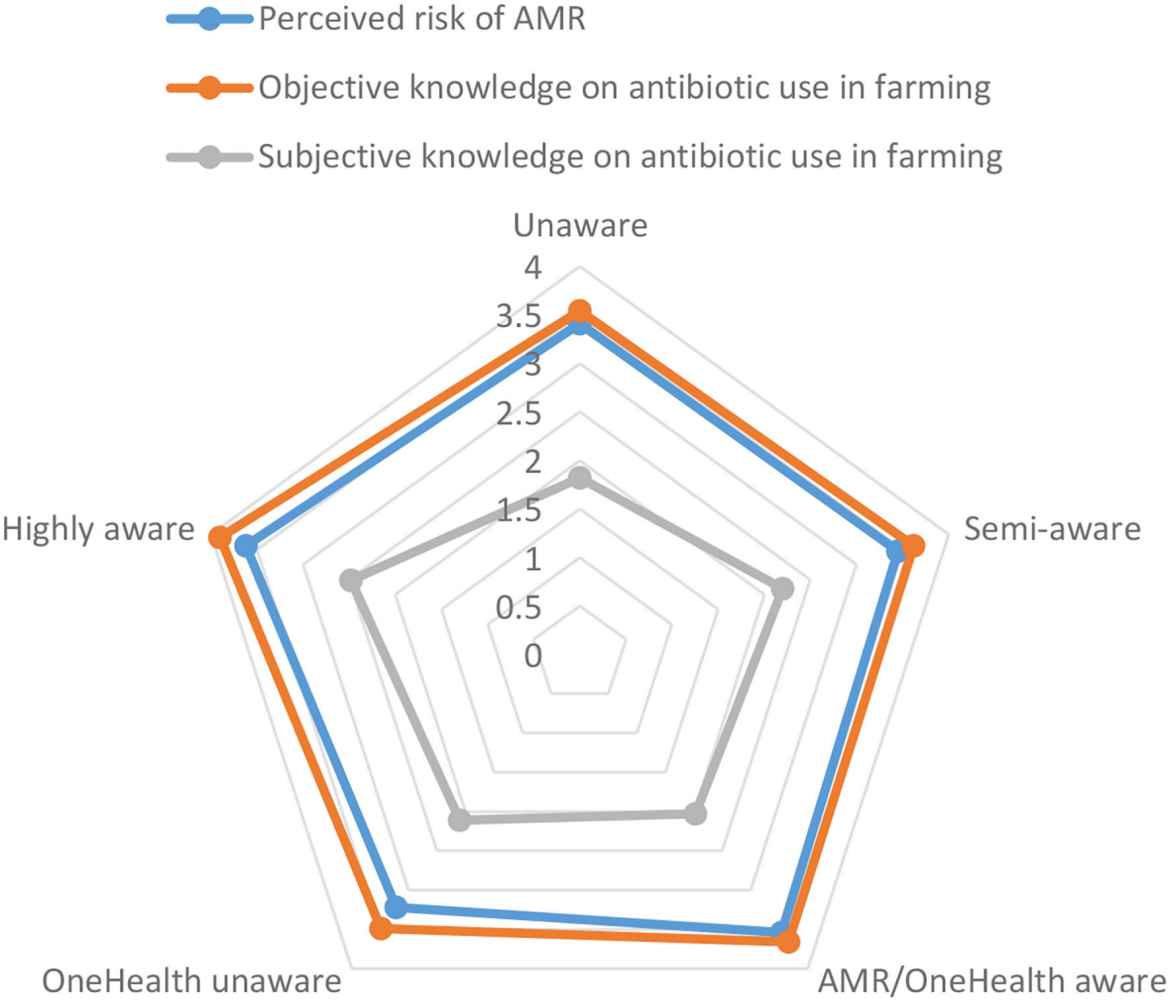
Agricultural antibiotic knowledge levels and AMR risk perceptions across five clusters of consumers, categorised based on perceived impact of Covid-19 on awareness of AMR, OneHealth, and welfare information on labels (*n* = 972).

**Figure 2:**
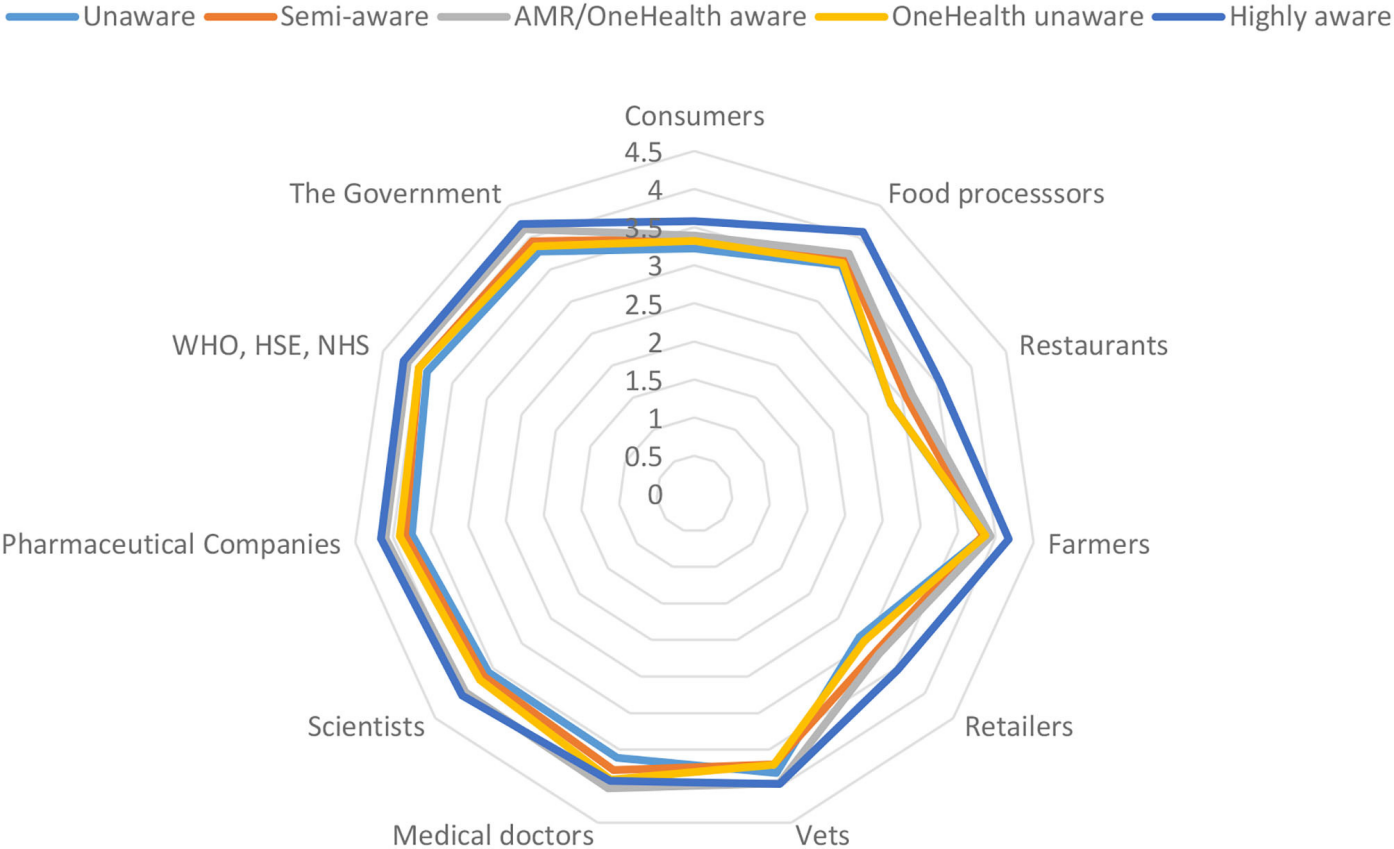
Attributions of responsibility for tackling AMR across five clusters of consumers, categorised based on perceived impact of Covid-19 on awareness of AMR, OneHealth, and welfare information on labels (*n* = 972).

Comparison of the five clusters showed they differed significantly on socio-demographic variables and on knowledge (objective and subjective) and beliefs (risk perception and responsibility attributions) related to antibiotic use in farming and AMR (Table 2). No significant association was detected between cluster membership and gender (*p* = 0.1584); age (*p* = 0.070); country (*p* = 0.057); urban-rural divide (*p* = 0.897); grandparent/relatives owning a farm (*p* = 0.591); and close neighbours/friends owning a farm (*p* = 0.859). Each segment profile is described in the following sections with results outlined in Table 2 and Figures 1, 2.

**Table 2:** Percentages or means (and standard deviations) of socio-demographic, knowledge, and belief variables across the five clusters.

	**Unaware segment**	**Semi-aware segment**	**AMR/OneHealth unaware segment**	**OneHealth unaware segment**	**Highly aware segment**	**Comparison of clusters with a *χ*^**2**^-test or ANOVA**
*Social class*						*χ*^2^ (4, *n* = 972) = 11.23, *p* = 0.024
ABC1F+	17.9%	32.1%	18.8%	10%	21.3%	
C2DEF–	14.9%	26.5%	23.2%	15.1%	20.3%	
*Living situation*						*χ*^2^ (4, *n* = 972) = 12.27, *p* = 0.015
Living alone	24.6%	31.9%	17.4%	13%	13%	
Living with others	15.2%	29.3%	21.2%	12.1%	22.2%	
*Parent of young child(ren)*						*χ*^2^ (4, *n* = 972) = 11.28, *p* = 0.024
Yes	16.2%	31.1%	22.3%	7.1%	23.3%	
No	16.7%	29%	20%	14.5%	19.8%	
*Parents have/had a farm*						*χ*^2^ (4, *n* = 972) = 15.20, *p* = 0.004
Yes	12.3%	22.2%	26.6%	15.8%	23.2%	
No	17.7%	31.6%	19.1%	11.3%	20.3%	
*Education^1^*	4.30 (1.07)^ab^	4.36 (1.00)^a^	4.02 (1.12)^b^	4.07 (1.17)^ab^	4.40 (0.95)^a^	*F*_(4,945)_ = 5.171, (*p* < 0.001)
*Objective knowledge antibiotic use in agriculture^2^*	3.54 (0.87)^a^	3.62 (0.94)^a^	3.66 (0.89)^a^	3.49 (0.77)^a^	3.90 (0.87)^b^	*F*_(4,967)_ = 5.901, (*p* < 0.001)
*Subjective knowledge antibiotic use in agriculture^3^*	1.82 (0.76)^a^	2.20 (0.86)^b^	2.03 (0.88)^ab^	2.11 (0.80)^b^	2.48 (0.99)^c^	*F*_(4,967)_ = 13.929, (*p* < 0.001)
*AMR risk perception^4^*	3.41 (0.84)^ab^	3.45 (0.76)^a^	3.54 (0.77)^a^	3.22 (0.73)^b^	3.62 (0.82)^a^	*F*_(4,967)_ = 5.635, (*p* < 0.001)
*Responsibility attributions for action on AMR^5^*						
Consumers	3.22 (1.18)^a^	3.35 (1.06)^ab^	3.39 (1.11)^ab^	3.32 (1.07)^ab^	3.58 (1.16)^b^	*F*_(4,967)_ = 2.641, (*p* = 0.033)
Food processors/manufacturers	3.57 (1.15)^a^	3.67 (0.97)^a^	3.75 (0.97)^a^	3.60 (1.12)^a^	4.09 (0.91)^b^	*F*_(4,967)_ = 8.267, (*p* < 0.001)
Restaurants/fast food chains/caterers	2.84 (1.13)^a^	3.06 (1.07)^a^	3.14 (1.06)^a^	2.84 (1.12)^a^	3.54 (1.09)^b^	*F*_(4,967)_ = 12.154, (*p* < 0.001)
Farmers	3.85 (1.01)^a^	3.83 (0.95)^a^	3.93 (0.86)^ab^	3.86 (0.93)^a^	4.17 (0.83)^b^	*F*_(4,967)_ = 4.721, (*p* = 0.001)
Retailers	2.87 (1.1)^a^	3.16 (1.04)^ab^	3.21 (1.09)^b^	2.95 (1.11)^ab^	3.53 (1.16)^c^	*F*_(4,967)_ = 9.605, (*p* < 0.001)
National government	3.78 (1.02)^a^	3.94 (0.92)^ab^	4.13 (0.85)^b^	3.86 (1.02)^ab^	4.21 (0.87)^bc^	*F*_(4,967)_ = 6.727, (*p* < 0.001)
Medical doctors	3.61 (1.20)^a^	3.78 (1.05)^ab^	4.03 (0.90)^b^	3.91 (1.00)^ab^	3.93 (1.05)^b^	*F*_(4,967)_ = 4.255, (*p* = 0.002)
Veterinarians	3.82 (1.08)^abc^	3.70 (1.00)^ab^	3.96 (0.98)^abc^	3.71 (1.00)^abc^	3.97 (1.09)^ac^	*F*_(4,967)_ = 3.238, (*p* = 0.012)
Scientists	3.58 (1.07)^a^	3.66 (1.05)^a^	3.98 (0.89)^b^	3.73 (1.16)^ab^	4.04 (0.91)^b^	*F*_(4,967)_ = 7.979, (*p* < 0.001)
Pharmaceutical companies	3.75 (1.12)^a^	3.84 (1.03)^ab^	4.10 (0.90)^c^	3.91 (0.95)^abc^	4.16 (0.96)^c^	*F*_(4,967)_ = 5.858, (*p* < 0.001)
Public organisations (e.g., NHS, HSE, WHO)	3.86 (1.13)^a^	3.98 (0.96)^abc^	4.14 (0.83)^bc^	3.98 (1.00)^abc^	4.20 (0.91)^c^	*F*_(4,967)_ = 3.758, (*p* = 0.005)

1 *Higher scores, higher level of education attained;*

2 *higher scores, higher knowledge;*

3 *higher scores, more subjective knowledge;*

4 *higher scores, higher perceived risk;*

5 *higher scores, higher perceived responsibility*.

*Means sharing a common superscript within a row are not significantly different, Tukey p < 0.05*.

### Unaware Segment

Participants in this segment accounted for 16% of the sample. Covid-19 had little impact on increasing their awareness of AMR, connected animal and human health, and animal welfare information on food labels. These participants were more likely to live alone. They were less likely to come from a farming family and perceived they have the least knowledge about the use of antibiotics in farming, signalling a detachment from farming practises. They had the lowest responsibility ratings for almost all stakeholder groups, indicating a lower likelihood of demanding change from any sector or stakeholder on AMR. From the perspective of communicating with the public about the use of antibiotics in farming, this group will be the hardest to reach. Labelling strategies on agricultural antibiotic use would likely be ineffective. For these types of consumers, and as a first step, there is a need to find a way to increase awareness and knowledge about AMR, OneHealth, and the role of responsible antibiotic use in farming. Given that Covid-19, as a OneHealth emergency in itself, failed to raise awareness, this is not likely to be an easy task.

### Semi-aware Segment

This was the largest segment (30%). Covid-19 did not increase participants' awareness of AMR and only moderately increased their awareness of connected animal and human health and welfare information on labels. These participants were more likely to be in a higher social class and have a higher level of education. Participants tended not to come from a farming family and compared to the other clusters, they were less likely to attribute responsibility for AMR to farmers and vets. Considering their profile, it could be that this group knows little about AMR and antibiotic use in agriculture. This segment may be unlikely to expect change from the farming community with respect to action on AMR. For this segment, representing almost a third of the sample, top-down labelling strategies that proactively communicate about responsible antibiotic use in farming may have little impact. What is first required is an engagement effort that makes clear the concept of connected human and animal health, links between agriculture and AMR, and the need for responsible agricultural antibiotic use.

### AMR/OneHealth Aware Segment

Participants in this cluster (21%) were characterised by an increase in awareness of both AMR and the connection between animal and human health but were not likely to increase their awareness of animal welfare information on labels. Participants were more likely to come from a lower social class, have a lower level of education, live with others and come from a farming family. Along with the “highly aware” cluster, this cluster tended to have higher levels of responsibility attribution overall. Their increased awareness and higher responsibility attributions indicate that participants see a need for action on AMR. However, compared to the other clusters, this group attributed more AMR responsibility to medical professionals—both to vets, and in particular, to medical doctors. It is potentially the case that participants in this segment do not make a connection between issues such as AMR and OneHealth and a need for behaviour change at an individual level (e.g., use of labels). It may be useful to raise awareness and motivation around the need for collective action across different types of stakeholders at all levels, including at the individual consumer level (e.g., consumption and purchasing decisions).

### OneHealth Unaware Segment

This was the smallest segment in the sample (12%). Covid-19 increased their awareness of AMR strongly, and of animal welfare information on labels moderately, but did not increase their awareness of connected animal and human health. Participants were more likely to be from a lower social class, come from a farming family, and not have a young child. These participants were least knowledgeable about antibiotic use in farming. They held the lowest AMR risk perceptions and tended to have lower levels of responsibility attribution for AMR overall, signalling that although their awareness of AMR may have increased, they did not view it as an issue of concern or warranting widespread action. In particular, they attributed less responsibility for AMR to agri-food stakeholders and more responsibility to stakeholders such as pharmaceutical companies, public health organisations, scientists, and in particular, medical doctors. Accordingly, this group may view AMR as a “human health” issue and be less aware of the OneHealth connection to AMR. These consumers may not understand the contribution of antibiotic use on farms to AMR, or be aware that AMR also can affect the health and welfare of animals. In such a low-knowledge environment, it is hard to anticipate how such consumers would react to antibiotic-use labels. However, given this group's awareness of farm animal welfare labels; they are likely to be at least interested in them. It would be important to ensure a better understanding of the role of responsible antibiotic use in farming for positive animal welfare.

### Highly Aware Segment

Accounting for one-fifth of the sample, these participants (20.88%) were the most heavily impacted by Covid-19. Their awareness of AMR, connected animal and human health, and animal welfare information on food labels had all strongly increased. Participants had a higher level of education, were more likely to live with others, be a parent of a young child, and come from a farming background. They were both the most knowledgeable about the use of antibiotics in farming, and they *perceived* that they had the most knowledge about antibiotic use in farming. They were the most concerned about AMR in respect of risk perception and they were the group most likely to attribute high levels of responsibility for tackling AMR to almost all stakeholders. Given their interest in and concern for AMR, and their awareness of the role of antibiotics in farming, this is a primed audience for immediate or future market-level strategies related to responsible antibiotic use in agriculture.

## Conclusion

Covid-19 has created additional challenges for tackling AMR—not least of which includes concerns over increased indiscriminate use of antibiotics to treat ill Covid-19 patients (3); but, it has also created opportunities (20). It provides a basis to build momentum amongst stakeholders to embrace collaborative actions to tackle OneHealth emergencies. This pandemic has served to make the abstract nature of the “OneHealth” concept a daily reality for many individuals and ensuring “collective responsibility” to combat Covid-19 has been a particularly resonant concept. This could have implications for how people view similar OneHealth issues such as AMR going forward. This is important; AMR is a “collective moral action problem” (21) which requires *all* stakeholders (e.g., vets, doctors, farmers, patients, food consumers) to change their behaviour so as to minimise the spread and development of AMR; more responsibly use antibiotics; or alleviate the need for antibiotics in the first place. At the consumer level, this equates to changing food purchasing and consumption practises (e.g., choosing food from farm systems with responsible antibiotic use practises). The current study provides early evidence that Covid-19 has catalysed increased awareness of AMR and OneHealth. There may be appetite amongst some consumers to support actions for responsible antibiotic use on farms. However, the pathway for consumer change is not straightforward. A number of conditions need to be met to ensure an effective and responsible market-level strategy.

This study shows that Covid-19 has served to catalyse awareness of AMR, OneHealth and animal welfare information on labelling amongst a specific category of consumers who as a result, are likely to be increasingly primed for market-level strategies that place a value on responsible antibiotic use (e.g., OneHealth logos, “responsible use” logos, or quality assurance marks). The current study provides an indication of likely support; further research exploring consumers' willingness-to-purchase would be of value (22). However, any market-level strategy that incorporates antibiotic-use labelling needs to ensure an environment of complete consumer awareness and understanding about the role of antibiotics in farming—both its' contribution to AMR, and the need for *responsible* (rather than eliminated) use of antibiotics at farm level. This is a fundamental condition for an effective labelling strategy, not only to motivate individual consumer action regarding food choices, but also to avoid any unintended impacts or fall-out from consumer confusion regards the meaning of antibiotic-use labels (e.g., “antibiotic free”) (10, 11). The current study identifies consumer segments with low awareness levels about agricultural antibiotic use practises and the role of agriculture in tackling AMR. Such consumers at present are unlikely to engage with any form of antibiotic use labels. There is a need to consider what actions beyond mere labelling may be required to minimise the detachment of many consumers from the realities of farming and agri-food production. Covid-19 also presents an opportunity to consider larger transformational changes through short supply chain initiatives that have not only proven resilient in the face of the Covid-19 crisis, but also work to reconnect food producers and food consumers (12). By whatever means, an effective market-level strategy will require thorough consumer understanding of and motivation for responsible antibiotic use in farming, as well as an understanding of the role of collective responsibility, and where the consumer sits in this, in tackling a OneHealth emergency such as AMR.

A limitation of the current study was that the survey only measured perceived increases in consumers' awareness, captured through self-report. The increased awareness of AMR, connected human and animal health, and animal welfare information on labelling could signal that the public are increasingly interested in and aware of the potential impact of transfer through the food chain and desire more assurance in food production. However, we do not have any data to support whether this increased awareness has resulted in behaviour change. Future research avenues include exploring changes in purchasing and consumption behaviour including the extent to which consumers may begin to increase their use of welfare-specific food labels or purchase products with welfare-specific value attributes.

## Data Availability Statement

The raw data supporting the conclusions of this article will be made available by the authors, without undue reservation.

## Ethics Statement

Ethical review and approval was not required for the study on human participants in accordance with the local legislation and institutional requirements. The patients/participants provided their written informed consent to participate in this study.

## Author Contributions

AR was responsible for survey design and over-seeing data collection and led data analysis and paper write-up. SS was responsible for survey design and over-seeing data collection and contributed to paper write-up, CM, TB, and MD advised on survey design and contributed to paper write-up. JH advised on data analysis and contributed to paper write-up. All authors contributed to the article and approved the submitted version.

## Conflict of Interest

The authors declare that the research was conducted in the absence of any commercial or financial relationships that could be construed as a potential conflict of interest.
