# Supplementary material for: The Impact of the Covid-19 Pandemic on Food Consumers' Awareness of Antimicrobial Resistance, OneHealth, and Animal Welfare Information on Food Labels
Source: Front Vet Sci. 2021 Jun 29;8:678509. doi: 10.3389/fvets.2021.678509 (PMC8276886; doi:10.3389/fvets.2021.678509)
Supplement: Supplementary file 1 [file Table_1.DOCX]

**Supplementary File 1**

Sample Characteristics (*n* = 972)

| Characteristic | *N* = 972 | |
| --- | --- | --- |
| *Age (years)* |  | |
| 18-24 | 65 (6.7%) | |
| 25-34 | 201 (20.7%) | |
| 35-44 | 240 (24.7%) | |
| 45-54 | 183 (18.8%) | |
| 55-64 | 151 (15.5%) | |
| 65+ | 132 (13.6%) | |
|  |  | |
| *Gender* |  | |
| Male | 446 (45.9%) | |
| Female | 526 (54.10%) | |
|  |  | |
| *Country of residence* |  | |
| Northern Ireland | 285 (29.3%) | |
| Republic of Ireland | 693 (70.7%) | |
|  |  | |
| *Area of residence* |  | |
| Urban City | | 212 (21.8%) |
| Urban Town | | 220 (22.6%) |
| Suburban | | 265 (27.3%) |
| Rural Village | | 118 (12.10%) |
| Rural Countryside | | 157 (16.2%) |
|  |  | |
| *Social Class ^a^* |  | |
| ABC1F+ | 549 (56.5%) | |
| C2DEF- | 423 (43.5%) | |
|  |  | |
| *Education status* |  | |
| Primary or no formal education | 9 (0.9%) | |
| Lower secondary | 61 (6.3%) | |
| Higher secondary | 205 (21.1%) | |
| Post Leaving Certificate/A-Level | 81 (8.3%) | |
| Third level | 594 (61.1%) | |
| Not stated | 22 (2.2%) | |
|  |  | |
| *Dietary Preferences* |  | |
| Vegetarian | 40 (4.1%) | |
| Partial vegetarian | 94 (9.7%) | |
| Meat eater | 817 (84.1%) | |
| Other dietary restrictions | 21 (2.2%) | |

^a^ Social class is categorised by the Central Statistics Office based on the level of skill and educational attainment of one’s occupation. ABC1F+ = Professional, managerial and technical; C2DEF- = Skilled or semi-skilled manual and unskilled.
