# Supplementary material for: The Impact of the Covid-19 Pandemic on Food Consumers' Awareness of Antimicrobial Resistance, OneHealth, and Animal Welfare Information on Food Labels
Source: Front Vet Sci. 2021 Jun 29;8:678509. doi: 10.3389/fvets.2021.678509 (PMC8276886; doi:10.3389/fvets.2021.678509)
Supplement: Supplementary file 2 [file Table_2.DOCX]

**Exploring Consumer Perceptions of Farming & Food Production on the Island of Ireland**

**Questionnaire and Data Dictionary**

**Informed Consent**

**Entry Page**

Welcome to our Exploring Consumer Perceptions of Farming and Food Production on the Island of Ireland survey! This survey takes on average 15 minutes to complete and we would really appreciate your views. We would like to know more about what consumers believe about how animals are being reared and produced in agriculture on the Island of Ireland. We also wish to discover the impacts of new information on these topics. The information we receive from the survey will help us understand which issues are important to consumers, and give insight into preferences on receiving information. This research is being carried out by scientists in Teagasc and in Queen’s University Belfast.

**Page Break**

**[iQ1]** I understand the purpose of this study

*Yes (1) / No (2)*

**Page Break**

All the information collected about you during the course of the research will be kept strictly confidential. You will not be able to be identified or identifiable in any reports or publications. Any data collected about you during the survey will be stored in accordance with the General Data Protection Regulation 2016/679. Data collected may be shared in an anonymous form to allow reuse by the research team and other third parties. These anonymised data will not allow any participants to be identified or identifiable.

**[iQ2]** I understand that the information I provide in this survey will be anonymous.

*Yes (1) / No (2)*

**Page Break**

Please note that by taking part in this survey you are providing consent for your data to be included in this research. You can withdraw from the survey at any time by closing the browser window and your data will not be included in the study. Data will only be used once you have completed the survey.

**[iQ3]** I understand that I can withdraw from this survey at any time by closing the browser window.

*Yes (1) / No (2)*

**Page Break**

**[iQ5]** I agree to take part in this survey

*Yes (1) / No (2)*

**Socio-Demographics**

**[QGender]. Page Break**

Are you:

- Male (1)
- Female (2)

**[QAge]. Page Break**

How old are you? Please type numbers only:

**[QAgeRange] - Market research agency categorisation**

- under 11 (1)
- 11-15 years (2)
- 15-17 years (3)
- 18-24 years (4)
- 25-34 years (5)
- 35-44 years (6)
- 45-54 years (7)
- 55-64 years (8)
- 65+ years (9)

| **Data Cleaning**  QAgeRange was re-coded to get rid of the 3 redundant/empty categories (under 11; 11-15 years; 15-17 years). Variable now contains the following 6 categories:   - 18-24 years (1) - 25-34 years (2) - 35-44 years (3) - 45-54 years (4) - 55-64 years (5) - 65+ years (6) |
| --- |

**[QCounty].** **Page Break**

In which of the following counties do you currently live? (Drop-down: 32 options)

**[QRegion] - Market research agency categorisation**

- Dublin (1)
- Rest of Leinster (2)
- Munster (3)
- Connacht (4)
- Ulster (part of ROI) (5)
- Northern Ireland (6)

| **Data Cleaning**  **[Country]** – New variable created with responses as per below table  Republic of Ireland (1)  Northern Ireland (2)   \| Dublin (1) \| Republic of Ireland (1) \| \| --- \| --- \| \| Rest of Leinster (2) \| Republic of Ireland (1) \| \| Munster (3) \| Republic of Ireland (1) \| \| Connacht (4) \| Republic of Ireland (1) \| \| Ulster (part of ROI) (5) \| Republic of Ireland (1) \| \| Northern Ireland (6) \| Northern Ireland (2) \| |
| --- | --- | --- | --- | --- | --- | --- | --- | --- | --- | --- | --- | --- |

**[Q20PetOwner]. Page Break**

Do you have pets at home?

- Yes (1)
- No (2)

**[Q21DietaryPreferences]. Page Break**

Which of the following describes your dietary preference? 5 Categories currently

- Vegetarian (1)
- Partial vegetarian (2)
- Meat eater (3)
- Vegan (4) *“Thank you for taking our survey. Unfortunately, you don't qualify for this particular study.”* – redundant
- Other, please specify (5)

| **Data Cleaning**  Q21DietaryPreferences was re-coded to (a) get rid of vegan category and (b) re-code ‘other’ responses as per below table   - Vegetarian (1) - Partial vegetarian (2) - Meat eater (3) - Dietary restrictions (e.g. gluten-free, diabetic) (4)  \|  \| *N* \| Re-code to: \| \| --- \| --- \| --- \| \| Coeliac \| 2 \| (4) Dietary restrictions \| \| Dairy free \| 1 \| (4) Dietary restrictions \| \| diabetic \| 1 \| (4) Dietary restrictions \| \| Eat anything \| 1 \| (3) Meat eater \| \| eat more fish \| 1 \| (2) Partial vegetarian \| \| flexitarian \| 1 \| (2) Partial vegetarian \| \| Flexitarian \| 1 \| (2) Partial vegetarian \| \| flextian \| 1 \| (2) Partial vegetarian \| \| Gf \| 1 \| (4) Dietary restrictions \| \| Gluten Free \| 1 \| (4) Dietary restrictions \| \| glutton free \| 1 \| (4) Dietary restrictions \| \| Health and organic where possible \| 1 \| (3) Meat eater \| \| I eat white meats chicken fish Turkey rabbit lamb ham bacon \| 1 \| (3) Meat eater \| \| Keto \| 2 \| (4) Meat eater \| \| normal \| 1 \| (3) Meat eater \| \| Normal Diet \| 1 \| (3) Meat eater \| \| Pescatarian, gluten free \| 1 \| (2) Partial vegetarian \| \| Pollotarian \| 1 \| (2) Meat eater \| \| Wheat free \| 1 \| (4) Dietary restrictions \|   **[DietaryPreferences_Binary] –** New variable created with responses as per below table   - Vegetarian or partial vegetarian (1) - Meat eater (2)  \| Vegetarian (1) \| Vegetarian or partial vegetarian (1) \| \| --- \| --- \| \| Partial vegetarian (2) \| Vegetarian or partial vegetarian (1) \| \| Meat eater (3) \| Meat eater (2) \| \| Dietary restrictions (4) \| Assign as System Missing \| |
| --- | --- | --- | --- | --- | --- | --- | --- | --- | --- | --- | --- | --- | --- | --- | --- | --- | --- | --- | --- | --- | --- | --- | --- | --- | --- | --- | --- | --- | --- | --- | --- | --- | --- | --- | --- | --- | --- | --- | --- | --- | --- | --- | --- | --- | --- | --- | --- | --- | --- | --- | --- | --- | --- | --- | --- | --- | --- | --- | --- | --- | --- | --- | --- | --- | --- | --- | --- | --- |

**[QChildrenAmount].** **Page Break**

How many children do you have? For this question please only answer the number of children you are a parent or legal guardian to. 7 categories currently:

- None (1)
- 1 (2)
- 2 (3)
- 3 (4)
- 4 (5)
- 5 (6)
- 6 or more (7)

| **Data Cleaning**  **[Parent]** – New variable created with responses as per below table  No children (1)  Has children (2)   \| None (1) \| No children (1) \| \| --- \| --- \| \| 1 (2) \| Has children (2) \| \| 2 (3) \| Has children (2) \| \| 3 (4) \| Has children (2) \| \| 4 (5) \| Has children (2) \| \| 5 (6) \| Has children (2) \| \| 6 or more (7) \| Has children (2) \| |
| --- | --- | --- | --- | --- | --- | --- | --- | --- | --- | --- | --- | --- | --- | --- |

**[QChildAgeRanger1-10]. Page Break**

Do you currently have any children living in your household? Select all that apply

- Yes, aged 0-12 months (0 – not selected / 1 – selected)
- Yes, aged 1-3 years (0 – not selected / 1 – selected)
- Yes, aged 4-6 years (0 – not selected / 1 – selected)
- Yes, aged 7-9 years (0 – not selected / 1 – selected)
- Yes, aged 10-12 years (0 – not selected / 1 – selected)
- Yes, aged 13-15 years (0 – not selected / 1 – selected)
- Yes, aged 16-18 years (0 – not selected / 1 – selected)
- Yes, aged over 18 years (0 – not selected / 1 – selected)
- No, my children do not live at home (0 – not selected / 1 – selected)
- No, I don't have any children (0 – not selected / 1 – selected)

| **Data Cleaning**  **[ParentYoungChildren]** – New variable created with responses as per below table  Parent of child aged 0-12 (1)  Not a parent of child aged 0-12 (2)   \| Yes, aged 0-12 months \| Parent of child aged 0-12 (1) \| \| --- \| --- \| \| Yes, aged 1-3 years \| Parent of child aged 0-12 (1) \| \| Yes, aged 4-6 years \| Parent of child aged 0-12 (1) \| \| Yes, aged 7-9 years \| Parent of child aged 0-12 (1) \| \| Yes, aged 10-12 years \| Parent of child aged 0-12 (1) \| \| Yes, aged 13-15 years \| Not a parent of child aged 0-12 (2) \| \| Yes, aged 16-18 years \| Not a parent of child aged 0-12 (2) \| \| Yes, aged over 18 years \| Not a parent of child aged 0-12 (2) \| \| No, my children do not live at home \| Not a parent of child aged 0-12 (2) \| \| No, I don’t have any children \| Not a parent of child aged 0-12 (2) \| |
| --- | --- | --- | --- | --- | --- | --- | --- | --- | --- | --- | --- | --- | --- | --- | --- | --- | --- | --- | --- | --- |

**[QMaritalStatus]. Page Break**

Are you:

- Single (1)
- In a relationship (but not living together) (2)
- Living with partner (3)
- Married (4)
- Widowed (5)
- Divorced (6)
- Separated (7)
- Prefer not to say (8)

**[QChiefEarner]. Page Break**

Are you the Chief Income Earner in your household? The Chief Income Earner is the person in your household with the largest income. If your income is equal to your others in your family/household, or you are living in a shared/rented accommodation consider yourself the chief income earner.

- Yes (1)
- No (2)

**[QChiefOccupation]. Page Break**

To which occupational group does the Chief Income Earner in your household belongs to? The Chief Income Earner is the person in your household with the largest income. If your income is equal to others in your family/household, or you are living in a shared/rented accommodation, consider yourself the chief income earner.

- Higher Managerial/Professional/Administrative (i.e. Established Doctor; Dentist; Psychologist; Solicitor; Board Director in a large organisation (200+ employees); Top level civil servant or Public service employee; Business owner (10+ employees); Chartered Accountant; Architect, Pharmacist, Engineer, Professor) (1)
- Intermediate Managerial/Professional/Administrative (i.e. Newly qualified (under 3 years) Doctor; Solicitor, Board director in a small organisation; Middle manager in large organisation; Principle officer in civil service or local government; Business owner (<10 employees) (2)
- Supervisory/Clerical/Junior Managerial or Professional/Administrative (i.e. Office worker with no staff; Student Doctor; Teacher, Nurse, Guard, Social Care Worker, Foreman with 25+ employees; Salesperson) (3)
- Student (4)
- Skilled manual worker (i.e. Pub or Bar worker; Skilled Bricklayer; Carpenter; Plumber; Electrician; Painter; Bus or Ambulance Driver; HGV driver; AA patrolman) (5)
- Semi or unskilled manual work (Manual worker; Apprentice to the skilled trade; Labourer; Cleaner; Shop assistant; Waiter/Waitress; Caretaker; Park keeper; non-HGV driver) (6)
- Casual worker – not in permanent employment (7)
- Housewife / Home-maker (8)
- Retired (9)
- Farmer 1-49 acres (10)
- Farmer 50+ acres (11)
- Unemployed or not working due to long-term sickness (12)

| **Data Cleaning**  [QChiefOccupation] was recoded to get rid of the 2 redundant categories (Farmer 1-49 acres (10); Farmer 50+ acres (11)). Variable now contains the following 10 categories:   - Higher Managerial/Professional/Administrative (i.e. Established Doctor; Dentist; Psychologist; Solicitor; Board Director in a large organisation (200+ employees); Top level civil servant or Public service employee; Business owner (10+ employees); Chartered Accountant; Architect, Pharmacist, Engineer, Professor) (1) - Intermediate Managerial/Professional/Administrative (i.e. Newly qualified (under 3 years) Doctor; Solicitor, Board director in a small organisation; Middle manager in large organisation; Principle officer in civil service or local government; Business owner (<10 employees) (2) - Supervisory/Clerical/Junior Managerial or Professional/Administrative (i.e. Office worker with no staff; Student Doctor; Teacher, Nurse, Guard, Social Care Worker, Foreman with 25+ employees; Salesperson) (3) - Student (4) - Skilled manual worker (i.e. Pub or Bar worker; Skilled Bricklayer; Carpenter; Plumber; Electrician; Painter; Bus or Ambulance Driver; HGV driver; AA patrolman) (5) - Semi or unskilled manual work (Manual worker; Apprentice to the skilled trade; Labourer; Cleaner; Shop assistant; Waiter/Waitress; Caretaker; Park keeper; non-HGV driver) (6) - Casual worker – not in permanent employment (7) - Housewife / Home-maker (8) - Retired (9) - Unemployed or not working due to long-term sickness (10) |
| --- |

**[QSocialClass2] - Market research agency categorisation**

- ABC1F+ (1)
- C2DEF- (2)

This variable (created by market research agency for sampling purposes) categorises “Social Class” according to profession with the two social classes organised as follows:

- A = High managerial, administrative or professional
- B= Intermediate managerial, administrative or professional
- C1 = Supervisory, clerical and junior managerial, administrative or professional
- C2 = Skilled manual workers
- D = Semi and unskilled manual workers
- E = State pensioners, casual or lowest grade workers, unemployed with state benefits only
- F+ = farmers with 50+ acres
- F- = Farmers with less than 50 acres

**[QEmploymentStatus]. Page Break**

What is your current employment status? If more than one option is applicable, e.g. employed part-time and student, please select the activity that takes up the most of your time.

- Working full-time (35 or more hours per week) (1)
- Working part-time (less than 35 hours per week) (2)
- Contract, Temporary or Freelance Employee (3)
- Self-employed full-time (4)
- Self-employed part-time (5)
- Unemployed and looking for work (6)
- Looking after my home/family full-time (7)
- Student (Full-time education, not working) (8)
- Student (Working less than 30hrs per week) (9)
- Student (Working more than 30hrs per week) (10)
- Retired (11)
- Unable to work (12)

**[Q18r1-3]. Page Break**

**Measures**: “Farm experience”

**Adapted from:** Vanhonachker, F., Verbeke, W., Van Poucke, E., and Tuyttens, F. A. M. (2007). Segmentation based on consumers' perceived importance and attitude toward farm animal welfare. *The* *International Journal of Sociology of Food and Agriculture, 15*(3): 91-107.

Please answer ‘yes’ or ‘no’ to each of the following statements:

My parents have / had a farm (Yes – 1 / No – 2)

My grandparents / other relatives have a farm (Yes – 1 / No – 2)

Close neighbours / good acquaintances have a farm (Yes – 1 / No – 2)

**[Q19FarmVisits]. Page Break**

**Measures:** “Farm visits”

**Adapted from:** Vanhonachker, F., Verbeke, W., Van Poucke, E., and Tuyttens, F. A. M. (2007). Segmentation based on consumers' perceived importance and attitude toward farm animal welfare. *The* *International Journal of Sociology of Food and Agriculture, 15*(3): 91-107.

During your lifetime, how many times have you visited a farm which rears livestock such as cows, pigs, chickens, sheep?

Never (1)

Once (2)

2-3 times (3)

4-5 times (4)

More than 5 times (5)

**[QEducation]. Page Break**

What is the highest level of education you have completed to date?

- Primary School Level (1)
- Lower Secondary (Inter/Junior Certificate) (2)
- Higher Secondary (Leaving Certificate) (3)
- Post Leaving Certificate (e.g. VEC) (4)
- Third Level Non-Degree (e.g. Diploma) (5)
- Undergraduate degree (less than 2 years) (6)
- Undergraduate degree (3 years) (7)
- Undergraduate degree (4 or more years) (8)
- Post graduate: Masters (9)
- Post graduate: PhD (10)
- Post graduate: other (non-Masters or PhD) (11)
- None of the above (12)
- Prefer not to say (13)

| **Data Cleaning**  **[EducationCSO] -** New variable created where QEducation was recoded according to Central Statistics Office categories, as per below  Primary or no formal education (1)  Lower secondary education (2)  Higher secondary education (3)  Post leaving certification (4)  Third level (5)  Not stated (6)   \| Primary School Level (1) \| Primary or no formal education (1) \| \| --- \| --- \| \| Lower Secondary (Inter/Junior Certificate) (2) \| Lower secondary education (2) \| \| Higher Secondary (Leaving Certificate) (3) \| Higher secondary education (3) \| \| Post Leaving Certificate (e.g. VEC) (4) \| Post leaving certification (4) \| \| Third Level Non-Degree (e.g. Diploma) (5) \| Third level (5) \| \| Undergraduate degree (less than 2 years) (6) \| Third level (5) \| \| Undergraduate degree (3 years) (7) \| Third level (5) \| \| Undergraduate degree (4 or more years) (8) \| Third level (5) \| \| Post graduate: Masters (9) \| Third level (5) \| \| Post graduate: PhD (10) \| Third level (5) \| \| Post graduate: other (non-Masters or PhD) (11) \| Third level (5) \| \| None of the above (12) 🡪 6 \| Not stated (6) \| \| Prefer not to say (13) 🡪 6 \| Not stated (6) \| |
| --- | --- | --- | --- | --- | --- | --- | --- | --- | --- | --- | --- | --- | --- | --- | --- | --- | --- | --- | --- | --- | --- | --- | --- | --- | --- | --- |

**[QHHShopper]. Page Break**

Which of the following describes your household responsibility for grocery shopping?

- I am solely responsible for grocery shopping for the household (1)
- I am partly responsible for grocery shopping for the household (2)
- I have no responsibility for grocery shopping (3)

| **Data Cleaning**  QHHShopper was re-coded to get rid of the redundant category (I have no responsibility for grocery shopping). Variable now contains the following 2 categories:   - I am solely responsible for grocery shopping for the household (1) - I am partly responsible for grocery shopping for the household (2) |
| --- |

**[QOccupants]. Page Break**

How many people live in the household including you?

- 1 (1)
- 2 (2)
- 3 (3)
- 4 (4)
- 5 (5)
- 6 or more (6)

| **Data Cleaning**  **[LivingSituation] -** New variable created where QOccupants was recoded to a binary variable indicating living situation  Living alone (1)  Living with others (2)   \| 1 (1) \| Living alone (1) \| \| --- \| --- \| \| 2 (2) \| Living with others (2) \| \| 3 (3) \| Living with others (3) \| \| 4 (4) \| Living with others (4) \| \| 5 (5) \| Living with others (5) \| \| 6 or more (6) \| Living with others (5) \| |
| --- | --- | --- | --- | --- | --- | --- | --- | --- | --- | --- | --- | --- |

**[QUrbanRural]. Page Break**

How would you describe where you live?

- Urban City (1)
- Urban Town (2)
- Suburban (3)
- Rural Village (4)
- Rural Countryside (5)

**[QIncome]. Page Break**

In which of the following bands would you place your personal yearly income (before tax and other deductions)?

- Less than €3,000 (1)
- €3,000 - €4,999 (2)
- €5,000 - €9,999 (3)
- €10,000 - €19,999 (4)
- €20,000 - €29,999 (5)
- €30,000 - €39,999 (6)
- €40,000 - €49,999 (7)
- €50,000 - €59,999 (8)
- €60,000 - €69,999 (9)
- €70,000 - €79,999 (10)
- €80,000 - €89,999 (11)
- €90,000 - €99,999 (12)
- €100,000 or more (13)
- Don't know / prefer not to say (14)

| **Data Cleaning**  **[PersonalIncomeCategories] -** New variable created where QIncome was recoded into categories  Low income (1)  Medium income (2)  High income   \| Less than €3,000 (1) \| Low income (1) \| \| --- \| --- \| \| €3,000 - €4,999 (2) \| Low income (1) \| \| €5,000 - €9,999 (3) \| Low income (1) \| \| €10,000 - €19,999 (4) \| Low income (1) \| \| €20,000 - €29,999 (5) \| Medium income (4) \| \| €30,000 - €39,999 (6) \| Medium income (4) \| \| €40,000 - €49,999 (7) \| Medium income (4) \| \| €50,000 - €59,999 (8) \| Medium income (4) \| \| €60,000 - €69,999 (9) \| Medium income (4) \| \| €70,000 - €79,999 (10) \| High income (3) \| \| €80,000 - €89,999 (11) \| High income (3) \| \| €90,000 - €99,999 (12) \| High income (3) \| \| €100,000 or more (13) \| High income (3) \| \| Don't know / prefer not to say (14) \| System missing \| |
| --- | --- | --- | --- | --- | --- | --- | --- | --- | --- | --- | --- | --- | --- | --- | --- | --- | --- | --- | --- | --- | --- | --- | --- | --- | --- | --- | --- | --- |

**[QHHIncome]. Page Break**

In which of the following bands would you place your household's combined yearly income (before tax and other deductions)? Household income is a measure of the combined incomes of all people sharing a particular household or place of residence. It includes every form of income, e.g. salaries and wages, retirement income, investment gains etc.

- Up to €10,000 (1)
- €10,000 - €19,999 (2)
- €20,000 - €29,999 (3)
- €30,000 - €39,999 (4)
- €40,000 - €49,999 (5)
- €50,000 - €59,999 (6)
- €60,000 - €69,999 (7)
- €70,000 - €79,999 (8)
- €80,000 - €89,999 (9)
- €90,000 - €99,999 (10)
- €100,000 - €109,999 (11)
- €110,000 - €119,999 (12)
- €120,000 - €129,999 (13)
- €130,000 - €139,999 (14)
- €140,000 - €149,999 (15)
- €150,000 or more (16)
- Don't know / prefer not to say (17)

| **Data Cleaning**  **[HouseHoldIncomeCategories] -** New variable created where HHIncome was recoded into categories  Low income (1)  Medium income (2)  High income   \| Up to €10,000 (1) \| Low income (1) \| \| --- \| --- \| \| €10,000 - €19,999 (2) \| Low income (1) \| \| €20,000 - €29,999 (3) \| Low income (1) \| \| €30,000 - €39,999 (4) \| Low income (1) \| \| €40,000 - €49,999 (5) \| Low income (1) \| \| €50,000 - €59,999 (6) \| Medium income (4) \| \| €60,000 - €69,999 (7) \| Medium income (4) \| \| €70,000 - €79,999 (8) \| Medium income (4) \| \| €80,000 - €89,999 (9) \| Medium income (4) \| \| €90,000 - €99,999 (10) \| Medium income (4) \| \| €100,000 - €109,999 (11) \| Medium income (4) \| \| €110,000 - €119,999 (12) \| High income (3) \| \| €120,000 - €129,999 (13) \| High income (3) \| \| €130,000 - €139,999 (14) \| High income (3) \| \| €140,000 - €149,999 (15) \| High income (3) \| \| €150,000 or more (16) \| High income (3) \| \| Don't know / prefer not to say (17) \| System missing \| |
| --- | --- | --- | --- | --- | --- | --- | --- | --- | --- | --- | --- | --- | --- | --- | --- | --- | --- | --- | --- | --- | --- | --- | --- | --- | --- | --- | --- | --- | --- | --- | --- | --- | --- | --- |

**Antibiotics**

**[Q. 10r1-6]. Page Break**

**Measures:** “Attitudes to antibiotic use in farming”

**Adapted from**: Frewer, L. J., Miles, S., and Marsh, R. (2002). The media and genetically modified foods: Evidence in support of social amplification of risk. Risk Analysis 22(4): 701-711.

Here are some statements others like you have made in relation to farming and food production. Please indicate how much you agree or disagree with each statement using the options provided.

|  | **Strongly Disagree (1)** | **Disagree (2)** | **Neither agree nor disagree (3)** | **Agree (4)** | **Strongly Agree (5)** |
| --- | --- | --- | --- | --- | --- |
| The use of antibiotics in farming leads to better quality food *[positive effects]* |  |  |  |  |  |
| The use of antibiotics in farming is important for good animal welfare *[positive effects]* |  |  |  |  |  |
| The use of antibiotics in farming makes me wonder what we’re eating *[negative effects]* |  |  |  |  |  |
| The use of antibiotics in farming will negatively affect future generations *[negative effects]* |  |  |  |  |  |
| The use of antibiotics in farming is important for protecting animal health *[positive effects]5* |  |  |  |  |  |
| The use of antibiotics in farming has negative effects on human health *[negative effects]* |  |  |  |  |  |

| **Data Cleaning**  **[ABinAgAttitudes] -** New variable created for Attitudes towards the use of antibiotics in agriculture where higher scores = more negative attitudes  Step 1: Reverse scored the 3 positive attitude items  Step 2: Sum the 6 attitude items from Q10 (Compute: SUM)  Step 3: Divide the total sum by 6 to give overall score (Compute: DIVIDE) |
| --- |

**[Q. 11_1-3]. Page Break**

**Measures:** “Awareness of antibiotic resistance”

**Adapted from:** Kosiyaporn, H., et al. (2020). Surveys of knowledge and awareness of antibiotic use and antimicrobial resistance in general population: a systematic review. *PLoS ONE, 15*(1): e0227973.

Please answer the following:

- I am aware of the issue of antibiotic resistance (Yes – 1 / No – 2)
- Antibiotic resistance is a problem in my country and worldwide (Yes – 1 / No – 2 / I don’t know - 3)
- Antibiotic resistance is an issue that could affect me or my family (Yes – 1 / No – 2 / I don’t know - 3)

| **Data Cleaning**  Scores reversed for ease of interpretation – Lower scores equals lower awareness  I am aware of the issue of antibiotic resistance (No – 1 / Yes – 2)  Antibiotic resistance is a problem in my country and worldwide (No – 1 / Yes – 2 / I don’t know - 3)  Antibiotic resistance is an issue that could affect me or my family (No – 1 / Yes – 2 / I don’t know - 3) |
| --- |

**[Q. 12_1-6 *and* Q12b_1-6]. Page Break**

**Measures:** “Objective knowledge: antibiotic use in agriculture”

**Adapted from:** Kosiyaporn, H., et al. (2020). Surveys of knowledge and awareness of antibiotic use and antimicrobial resistance in general population: a systematic review. *PLoS ONE 15*(1): e0227973; and, McNulty, C., et al. (2007). Don't wear me out - the public's knowledge of and attitudes to antibiotic use. *Journal of Antimicrobial Chemotherapy, 59*: 727-738.

To the best of your knowledge, do you think these statements are true or false?

*[each item immediately followed by certainty question]*

|  | | | Based on your answer that [*Antibiotics can kill bacteria]* is [*True]*, how certain are you that the answer you gave is correct? | | | | |
| --- | --- | --- | --- | --- | --- | --- | --- |
|  | True (1) | False (2) | Very unsure (1) | Quite unsure (2) | Slightly unsure (3) | Quite sure (4) | Very sure (5) |
| Antibiotics can kill bacteria *[action]* | x |  |  |  |  |  |  |
| Antibiotics can kill viruses *[action]* |  | x |  |  |  |  |  |
| Overuse of antibiotics makes them become ineffective *[use]* | x |  |  |  |  |  |  |
| If you feel well after half the treatment, you can end a prescribed course of antibiotics *[use]* |  | x |  |  |  |  |  |
| Antibiotic resistance happens when bacteria become immune to antibiotics *[resistance]* | x |  |  |  |  |  |  |
| Antibiotic resistance happens when a person or animal becomes immune to antibiotics *[resistance]* |  | x |  |  |  |  |  |

| **Data Cleaning**  **[Q12_1_recoded – Q12_6_recoded] –** 6 new variables created where ‘true’ and ‘false’ answers where recoded to ‘incorrect’ (1) and ‘correct’ (2) as appropriate for each knowledge question. This allows for easier interpretation of each of the individual knowledge questions, if they need to be used on their own.  **[AMRObjectiveKnowledge] –** new variable created which gives each participant an overall score for their Objective Knowledge about AMR which takes into account their answer and their certainty about the answer they gave. Higher scores = higher overall objective knowledge about AMR  Step 1: Using associated Syntax file, 6 new variables created **[Q12_1T - Q12_6T]** which combine the answer and certainty score given for each knowledge question:  Incorrect answer = 0  Correct answer + very uncertain = 1  Correct answer + quite uncertain = 2  Correct answer + slightly uncertain = 3  Correct answer + quite certain = 4  Correct answer + very certain = 5  Step 2: Sum the 6 new knowledge variables (Q12_1T - Q12_6T) (Compute: SUM)  Step 2: Divide the total sum by 6 to give overall score (Compute: DIVIDE) |
| --- |

**[Q. 13r1-r4]. Page Break**

**Measures:** “Subjective knowledge: antibiotic use in agriculture”

**Adapted from:** Vanhonachker, F., Verbeke, W., Van Poucke, E., and Tuyttens, F. A. M. (2007). Segmentation based on consumers' perceived importance and attitude toward farm animal welfare. *The* *International Journal of Sociology of Food and Agriculture, 15*(3): 91-107

Please indicate how much you agree or disagree with each statement using the options provided:

|  | Strongly Disagree (1) | Disagree (2) | Neither agree nor disagree (3) | Agree (4) | Strongly Agree (5) |
| --- | --- | --- | --- | --- | --- |
| Compared to the average person, I know a lot about how antibiotics are used in farming |  |  |  |  |  |
| I have a lot of knowledge about antibiotic use in farming |  |  |  |  |  |
| My friends consider me as an expert on the use of antibiotics in farming |  |  |  |  |  |
| I feel like I have enough knowledge about the topic of antibiotic use in farming |  |  |  |  |  |

| **Data Cleaning**  **[ABinAgSubjectiveKnowledge] -** New variable created for Subjective Knowledge about antibiotics in agriculture where higher scores = high subjective knowledge  Step 1: Sum the 4 items from Q13 (Compute: SUM)  Step 2: Divide the total sum by 4 to give overall score (Compute: DIVIDE) |
| --- |

**[Q. 14_1-6 *and* Q14b_1-6]. Page Break**

**Measures:** “Objective knowledge: antibiotic use in agriculture”

**Adapted from:** Etienne, J., et al. (2017). EU Insights – Perceptions on the human health impact of antimicrobial resistance (AMR) and antibiotics use in animals across the EU. EFSA supporting publication. Parma, Italy, European Food Safety Authority; and, Alexa, E. A., et al. (2019). A European questionnaire-based study on population awareness and risk perception of antimicrobial resistance. FEMS Microbiology Letters, 366(17): fnz221.

To the best of your knowledge, do you think these statements are true or false?

*[each item immediately followed by certainty question]*

|  | | | Based on your answer that [*Antibiotics are used to treat infections in farm animals]* is [*True]*, how certain are you that the answer you gave is correct? | | | | |
| --- | --- | --- | --- | --- | --- | --- | --- |
|  | True (1) | False (2) | Very unsure (1) | Quite unsure (2) | Slightly unsure (3) | Quite sure (4) | Very sure (5) |
| Antibiotics are used to treat infections in farm animals *[use]* | x |  |  |  |  |  |  |
| Antibiotics are used to prevent infections in farm animals *[use]* | x |  |  |  |  |  |  |
| The antibiotics used on farm animals are different from those used on people *[use]* |  | x |  |  |  |  |  |
| Under EU law, farmers are not allowed to use antibiotics to stimulate the growth of farm animals *[use]* | x |  |  |  |  |  |  |
| Overuse of antibiotics in farming makes them become ineffective to treat animals *[resistance]* | x |  |  |  |  |  |  |
| Bacteria resistant to antibiotics in farm animals can be transferred to people *[resistance]* | x |  |  |  |  |  |  |

| **Data Cleaning**  **[Q14_1_recoded – Q14_6_recoded] –** 6 new variables created where ‘true’ and ‘false’ answers where recoded to ‘incorrect’ (1) and ‘correct’ (2) as appropriate for each knowledge question. This allows for easier interpretation of each of the individual knowledge questions, if they need to be used on their own.  **[ABinAgObjectiveKnowledge] –** new variable created which gives each participant an overall score for their Objective Knowledge about Antibiotics in Agriculture which takes into account their answer and their certainty about the answer they gave. Higher scores = higher overall objective knowledge about antibiotics in agriculture  Step 1: Using associated Syntax file, 6 new variables created **[Q14_1T - Q14_6T]** which combine the answer and certainty score given for each knowledge question:  Incorrect answer = 0  Correct answer + very uncertain = 1  Correct answer + quite uncertain = 2  Correct answer + slightly uncertain = 3  Correct answer + quite certain = 4  Correct answer + very certain = 5  Step 2: Sum the 6 new knowledge variables (Q14_1T - Q14_6T) (Compute: SUM)  Step 2: Divide the total sum by 6 to give overall score (Compute: DIVIDE) |
| --- |

**Page Break**

Please read this explanation of the term ‘antibiotic resistance’:

Antibiotics are used to kill bacteria. They are an important medicine for treating infections in humans and animals. However, the more antibiotics are used, the more ineffective they become at killing the harmful bacteria. This is known as *antibiotic resistance*. You might also have heard of it as *antimicrobial resistance* (‘AMR’). Antibiotic resistance means illnesses in both human and animals are much harder to treat.

**[Q 15r1-r3]. Page Break**

**Measures:** “AMR risk perception”

**Adapted from:** Frewer, L. J., Shepherd, R., and Sparks, P. (1994). The Interrelationship between Perceived Knowledge, Control and Risk Associated with a Range of Food-Related Hazards Targeted at the Individual, Other People and Society. Journal of Food Safety 14(1): 19-40.

How do you perceive each of the following statements?-

|  | Very low (1) | Low (2) | Neutral (3) | High (4) | Very high (5) |
| --- | --- | --- | --- | --- | --- |
| The risks associated with antibiotic resistance to me personally are… |  |  |  |  |  |
| The risks associated with antibiotic resistance to the average person are… |  |  |  |  |  |
| The risks associated with antibiotic resistance to society are… |  |  |  |  |  |

| **Data Cleaning**  **[AMRRiskPerception] -** New variable created for Risk Perception about AMR where higher scores = higher risk perception  Step 1: Sum the 3 items from Q15 (Compute: SUM)  Step 2: Divide the total sum by 3 to give overall score (Compute: DIVIDE) |
| --- |

**[Q. 16r1-r11]. Page Break**

**Measures:** “Attributions of responsibility”

**Adapted from:** Regan, Á., Shan, L. C., Wall., P., and McConnon, Á. (2016). Perspectives of the public on reducing population salt intake in Ireland. Public Health Nutrition 19(7): 1327-1335.

How much responsibility do you believe lies with each of the following groups to take action to reduce the risk of antibiotic resistance? -

|  | Not at all responsible (1) | Slightly responsible (2) | Moderately responsible (3) | Very responsible (4) | Extremely responsible (5) |
| --- | --- | --- | --- | --- | --- |
| Consumers |  |  |  |  |  |
| Food processors / manufacturers |  |  |  |  |  |
| Restaurants / fast food chains / caterers |  |  |  |  |  |
| Farmers |  |  |  |  |  |
| Retailers |  |  |  |  |  |
| The national government |  |  |  |  |  |
| Medical doctors |  |  |  |  |  |
| Veterinarians |  |  |  |  |  |
| Scientists |  |  |  |  |  |
| Pharmaceutical companies |  |  |  |  |  |
| Public organisations (e.g. NHS, HSE, WHO) |  |  |  |  |  |

**[Q. 17r1-r3]. Page Break**

**Measures:** “Perceived impact of Covid-19 Pandemic”

**Adapted from:** New items developed for this survey.

Please rate your level of agreement with the following statements.

Compared to before the COVID-19 Pandemic…

|  | Strongly Disagree (1) | Disagree (2) | Neither agree nor disagree (3) | Agree (4) | Strongly Agree (5) |
| --- | --- | --- | --- | --- | --- |
| …I am now more aware of antibiotic resistance |  |  |  |  |  |
| …I look more at the labelling on food products for animal welfare information |  |  |  |  |  |
| …I am now more aware of the connection between the management of animal health and impact on human health |  |  |  |  |  |

**Debriefing**

**Page Break**

Finally, we would like to give you some information on this project.

**Page Break**

Due to the topic of the survey I would like to assure you that Irish agriculture and Irish farmers operate to a high standard of animal welfare. The welfare of farm animals, in Ireland, is highly regulated through the Department of Agriculture, Food & the Marine (DAFM) cross compliance inspection system. Furthermore, Bord Bia operate quality assurance schemes for the food industry. These schemes ensure best practice in farming and processing by adherence to relevant industry guidelines and international standards.

**Antimicrobial Resistance**

We asked you a number of questions during the survey, related to your knowledge of antibiotic resistance and antibiotic use on farms, the correct answer to each of these questions are as follows:


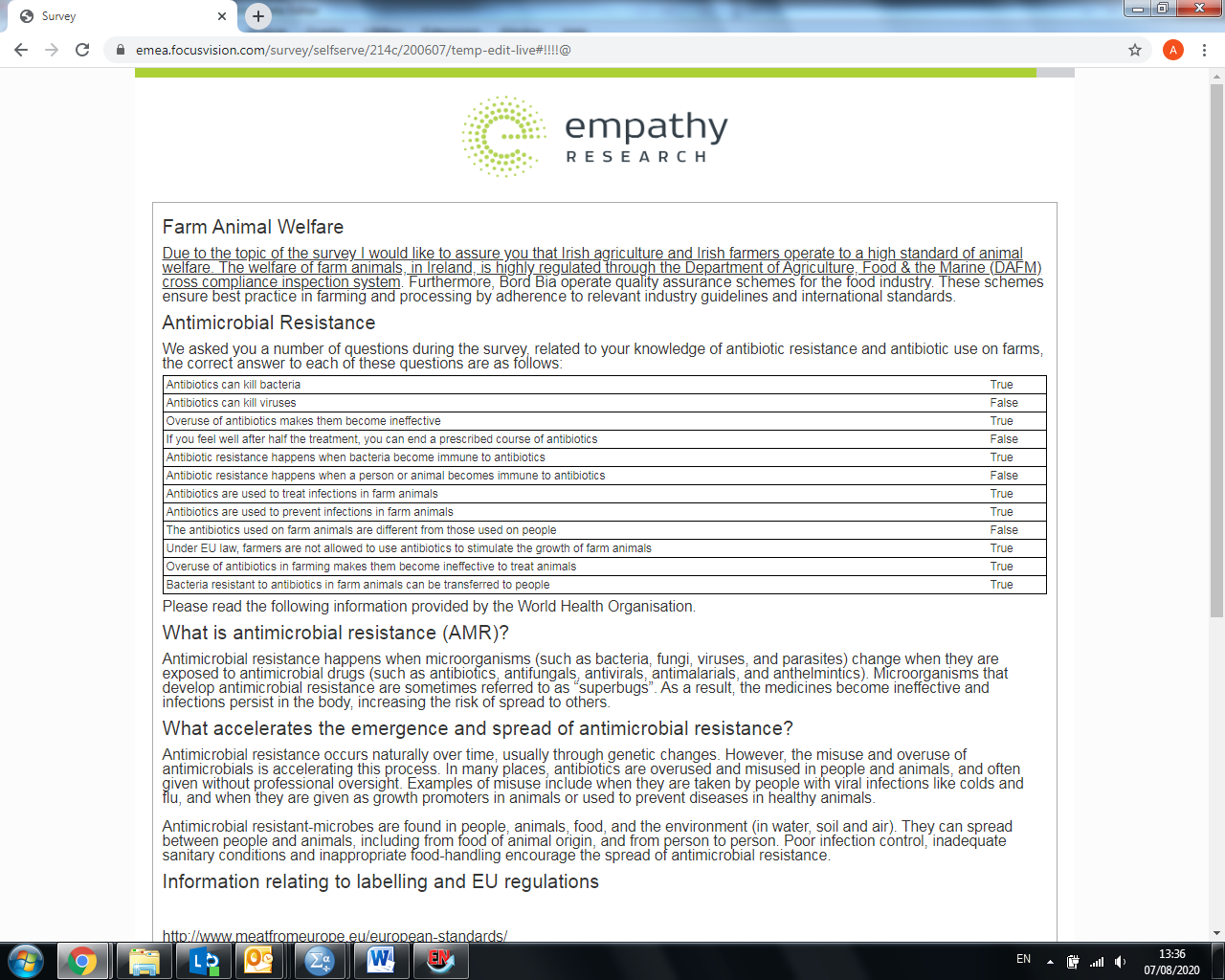


Please read the following information provided by the World Health Organisation.

**What is antimicrobial resistance (AMR)?**

Antimicrobial resistance happens when microorganisms (such as bacteria, fungi, viruses, and parasites) change when they are exposed to antimicrobial drugs (such as antibiotics, antifungals, antivirals, antimalarials, and anthelmintics). Microorganisms that develop antimicrobial resistance are sometimes referred to as “superbugs”. As a result, the medicines become ineffective and infections persist in the body, increasing the risk of spread to others.

**What accelerates the emergence and spread of antimicrobial resistance?**

Antimicrobial resistance occurs naturally over time, usually through genetic changes. However, the misuse and overuse of antimicrobials is accelerating this process. In many places, antibiotics are overused and misused in people and animals, and often given without professional oversight. Examples of misuse include when they are taken by people with viral infections like colds and flu, and when they are given as growth promoters in animals or used to prevent diseases in healthy animals. Antimicrobial resistant-microbes are found in people, animals, food, and the environment (in water, soil and air). They can spread between people and animals, including from food of animal origin, and from person to person. Poor infection control, inadequate sanitary conditions and inappropriate food-handling encourage the spread of antimicrobial resistance.

**Information relating to labelling and EU regulations**

<http://www.meatfromeurope.eu/european-standards/>

If you have any concerns or would like more information, please visit the sites linked below or contact a member of the research team.

Web links for further information

#### Websites for consumer information:

- Food safety Authority of Ireland: www.fsai.ie
- *safe*food Ireland: www.safefood.eu

#### Website for further information on farm animal health & welfare:

- DAFM: www.agriculture.gov.ie/animalhealthwelfare/
- Animal Welfare: [www.fawac.ie](http://www.fawac.ie)

#### Websites for further information on antimicrobial resistance:

- www.fsai.ie/faq/antimicrobial_resistance.html
- https://www.who.int/en/news-room/fact-sheets/detail/antibiotic-resistance
- https://www.who.int/news-room/facts-in-pictures/detail/antimicrobial-resistance
- https://www.who.int/foodsafety/areas_work/antimicrobial-resistance/amrfoodchain/en/

#### Contacts for further information

Dr Áine Regan, Agrifood Business and Spatial Analysis, Rural Economy & Development Programme, Teagasc. Email: [aine.regan@teagasc.ie](mailto:aine.regan@teagasc.ie)

Professor Moira Dean, School of Biological Sciences, Queen’s University Belfast, Northern Ireland. Email: [moira.dean@qub.ac.uk](mailto:moira.dean@qub.ac.uk)

**Final Page**

EU law places restrictive provisions on antibiotics, which may only be used for the treatment of herds and flocks under close supervision of a veterinarian. In the past, antibiotics were routinely added to animal feed as growth promoters, however, this practice was banned in the EU in 2006. For meat, eggs or milk to be sold in the EU, the animal has to undergo a ‘withdrawal period’ allowing antibiotics (and other drugs) to be processed and excreted, before animal produce can enter the food chain. Food has to comply with Maximum antibiotic Residue Limits (MRLs), set by the European Medicines Agency. These MRLs are considered to be safe for human consumption and below a concentration that inhibits bacteria. This is audited by the Veterinary Medicines Directorate and animal produce containing residues above the MRL are discarded and an investigation instigated. Consumers can be sure that they are eating meat that is completely safe and free of antibiotics, therefore, despite the use of antibiotics during production, the meat product does not contain antibiotics due to adherence with EU law and the appropriate withdrawal periods.

I have read the information provided relating to labelling and EU regulations. I understand that there are laws in place and that for meat, eggs or milk to be sold in the EU, the animal has to undergo a ‘withdrawal period’ allowing antibiotics (and other drugs) to be processed and excreted, before animal produce can enter the food chain.

**[QFinalQuestion_DebriefCheck]**

Please tick the box to indicate that you have read the above information:

- Yes (1)
- No (2)
